# Comparative Evaluation of the Accuracy of Electronic Apex Locator and Digital Radiography for Working Length Determination in Primary Teeth: A Systematic Review

**DOI:** 10.30476/dentjods.2023.97323.2006

**Published:** 2024-09-01

**Authors:** Ishita Agrawal, Farhin Katge, Sanmati Pol, Devendra Patil, Vamsi Krishna Chimata, Debapriya Pradhan

**Affiliations:** 1 Postgraduate Student, Dept. of Pediatric and Preventive Dentistry, Terna Dental College, Sector-22, Nerul West, Navi-Mumbai, India; 2 Dept. of Pediatric and Preventive Dentistry, Terna Dental College, Sector-22, Nerul West, Navi-Mumbai, Maharashtra, India; 3 Lecturer, Dept. of Pediatric and Preventive Dentistry, Terna Dental College, Sector-22, Nerul West, Navi-Mumbai, Maharashtra, India; 4 Reader, Dept. of Pediatric and Preventive Dentistry, Terna Dental College, Sector-22, Nerul West, Navi-Mumbai, Maharashtra, India

**Keywords:** Dental Radiovisiography, Digital Dental Radiography, Primary Dentitions, Milk Tooth

## Abstract

**Statement of the Problem:**

It is challenging to perform a pulpectomy procedure in primary tooth because of its physiological root resorption and variation in root morphology. Working length measurement is considered to be one of the critical steps, as it determines the extent of obturation and apical seal.

**Purpose:**

To compare the accuracy of electronic apex locator (EAL) and digital radiography (DR) for working length determination in primary teeth.

**Materials and Method:**

In this systematic review, electronic databases and grey literature were searched from 1st January 2005 to 1^st^ January 2023 for randomized control trial, non- randomized control trial, *in vitro* studies, *ex vivo* studies that compared accuracy of EAL and DR in primary teeth. Two reviewers independently identified studies, retrieved data, and assessed risk of bias using the revised and validated MINORS (methodological index for non-randomized studies) criteria.

**Results:**

Ten studies were included in qualitative analysis. Seven out of ten studies showed low risk of bias whereas other three studies showed high risk of bias. In view of methodological heterogeneity of the findings, a meta-analysis was not conducted.

**Conclusion:**

Available evidence suggests a moderate quality of evidence in this systematic review. Analyzing the ten studies included in this systematic review, the majority of studies showed statistically insignificant difference between EAL and DR. However, EAL was closer to actual WL as compared to DR. Based on the evidence that is currently available; EAL can be considered as an alternative for working length measurement in primary teeth.

## Introduction

Pediatric endodontic procedure helps in maintaining teeth in dentition, until their normal exfoliation time [ 1 ]. An accurate evaluation of root canal length determines the success of an endodontic procedure.

American Academy of Endodontics (2003) defined working length as ‘the distance from a coronal reference point to the point at which canal preparation and filling should terminate’ [ 2 ]. Accurate working length (WL) determination is extremely important, as it has an impact on ideal canal preparation, disinfection, and apical seal of root canal system [ 3 ]. The correct root canal length of primary teeth is difficult to predict because of the root resorption pattern, which could be either physiological or pathological. There is continuous alteration in size, shape, and position of root apices of primary teeth, [ 4 ] that causes difficulty in accurate determination of root canal length [ 5 ]. Traditional methods for establishing WL include conventional radiography, tactile sensation, moisture on a paper point, and knowledge of root canal anatomy.

After the introduction of digital radiography (DR), the first commercial integrated digital imaging system in dentistry was radiovisiography (RVG) which comprises an intraoral sensor instead of the conventional X-ray film [ 6 ]. DR is based on digital image capture and uses a charge-coupled device [ 3 ]. The advantages of a digital radiography above the conventional method are; predominantly a faster image procurement, lower radiation dose and image editing ability [ 7 ]. Although both conventional and DR methods offer some advantages, such as direct observation of root canal anatomy, presence of any periapical lesion and canal curvatures, there are limitations associated with them, such as radiation exposure and image distortion, which results in difficulty in identification of resorbed root apices [ 3 ]. Electronic apex locators (EAL) have been introduced to overcome the disadvantages of the above techniques. It is used in dentistry to determine where the apical constriction is located in the root canal [ 8 ]. It is a more accurate, easy, and painless technique, which is very useful in uncooperative children. Various advantages of EAL include; lesser radiation dosage as well as procedure time, which helps in maintaining patient cooperation. The efficacy of EAL has been proven even in the presence of root resorption, which is frequently encountered in primary teeth [ 9 ]. Considering the importance of WL determination for pediatric endodontic treatment and its maintenance until normal exfoliation, the aim of this systematic review was to compare the accuracy of EAL and DR for WL determination in primary teeth.

## Materials and Method

### Protocol registration and review reporting

The present systematic review was registered with protocol ID CRD42020222326 at the International Prospective Register of Systematic Reviews (PROSPERO). This systematic review was reported according to Preferred Reporting Items for Systematic Reviews and Meta-Analyses checklist (PRISMA 2020) [ 10 ]. 

### Research question

Research question for this systematic review was structured in PICOS format (participant, intervention, comparison, outcome and study design), depicted in Table 1.

**Table 1:** Population Intervention Comparison Outcome Study design (PICOS); EAL: Electronic apex locator, RVG: Radiovisiography, WL: Working length

Population	Human Primary Teeth
Intervention	EAL
Comparison	Radiovisiography (RVG)
Outcome	Working length (WL)
Study design	Randomized control trial, non-randomized control trial, *in vitro* studies, *ex vivo* studies.

Is there a difference in accuracy between an EAL and DR for WL determination in primary teeth? 

### Eligibility criteria

The eligibility criteria (Table 2) in this study were formulated to find studies based on the PICOS format. All studies were chosen in accordance with the criteria specified in this review.

**Table 2:** Eligibility criteria for qualitative assessment of studies (based on PICOS)

Inclusion Criteria	Exclusion Criteria
• Randomized control trial, non-randomized control trial, *in vitro* studies, *ex vivo* studies	Case reports, case series, reviews, book chapters, expert opinion, animal studies
• Studies performed on primary teeth	Articles reporting medically compromised patients
• Studies comparing electronic apex (EAL) and radiovisiography (RVG) to evaluate working length	Only abstracts
• Well defined information on working length using EAL and radiovisiography (RVG)	
• Articles published until 31^st^ August 2021	
• Articles published in English or which can be translated into english	

### Information sources and search strategy

Databases used in the search strategy included; Cochrane Library, National Library of Medicine (MEDLINE PubMed), Google scholar, EBSCOhost, Open grey literature, including both electronic and printed literature. The concept table (Table 3) denotes the terms used for search strategy, which included key concepts, as well as free text terms. Boolean operators such as OR/AND were used to combine search terms with other keywords relating to the review's goal. Additional articles were searched by looking through the references of the chosen publications, as well as previously published reviews on the topic, textbooks and publications, that met the inclusion criteria of the study.

**Table 3:** Concept table

	Population	Intervention	Comparison	Outcome
Key concept	Human primary teeth	Electronic apex locator	Radiovisiography	Working length determination
Free terms / Text words / TIAB terms	Deciduous dentition	Apex locators	Digital radiography	Working length
	Deciduous teeth	Formatron D10	Digital radiovisiography	Working length measurement
	Deciduous molar	Root Zx	Radiography	Apex localization
	Primary teeth	DentaPort ZX	Dental digital radiography	Root length determination
	Baby teeth	Propex II	Digital dental radiography	Root canal length
	Milk teeth	Root Zx Mini		
	Primary dentition	COXO C Smart-1 PRO		
	Primary molars	iPex.		
		Apex ID		
MeSH terms	Tooth		Radiography	
	Deciduous		Dental	
			Digital	

Articles published or studies conducted from January 1, 2005 to January 31, 2023 were included for this systematic review.

### Study selection

Selection of a study was done in three stages. In stage one, assessment of all the titles of the studies obtained through search strategy were done by two independent reviewers (IA and SP). Stage 2 involved screening of the abstracts, which was followed by screening of the full texts of relevant studies in stage 3. In case of disagreements between the two reviewers, a third reviewer (FK) was called in for a final decision. Only studies with full text reports were examined in this systematic review. Due to differences in the data supplied in abstract and those provided in the final report [ 11 ], literature published only as abstracts were excluded from the study. Authors were contacted for a full text of the relevant abstracts wherever possible. 

### Data collection and data items extracted

After defining the inclusion criteria for the selected articles, data extraction was performed independently by two review authors (IA and SP). Any disagreements in data extraction from the selected studies were discussed and resolved by a third (FK) and fourth (DP) reviewer. This information included authors name, year of publication, number of teeth included, demographic details of the patients (age in years), intervention, comparison, outcomes, study design and results.

### Study of risk of bias assessment

The risk of bias assessment was performed by two independent reviewers (IA & SP); using a modified version of the methodological items for nonrandomized studies (MINORS) scale (Annexure 1) [ 12 ]. A third reviewer (FK) validated all modifications to the MINORS scale. Cohen kappa coefficient for overall inter reviewer reliability was 0.725, indicating moderate agreement [ 13 ]. Any disagreements regarding risk of bias assessment, between the two reviewers (IA & SP), were resolved by a consensus between third (FK) and fourth (DP) reviewer. The items in MINORS scale were scored 0 (if not reported), 1 (if reported but inadequate) or 2 (if reported but adequate). The ideal score for comparative studies is 24. The checkpoints 6, 7 and 10 were excluded, as it was not applicable for this review. Hence, for the present systematic review 9 check-points were considered. The total score considered for the present review was 18. The score reported between 1 and 11 indicated high risk of bias, whereas score between 12 and18 indicated low risk of bias.

### Outcomes and data synthesis

The mean differences and their standard deviations were extracted and used in the presentation of results. The meta-analysis was not performed due to a high degree of heterogeneity seen in data extraction from different studies and methodologies.

## Results

### Study selection

**Figure 1:**
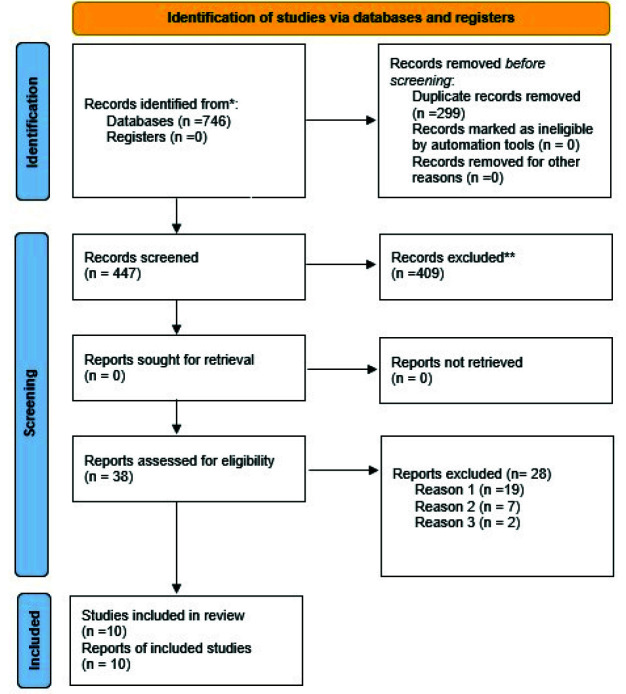
PRISMA 2020 flow diagram of the study

PRISMA flowchart (2020) describing the process of selecting studies is shown in Figure 1 (Page *et al*. 2020). Electronic literature search yielded 746 results. In the initial step of the screening process, duplicates were removed (299) using Mendeley software for Windows (Mendeley Ltd, Version 1803 Elsevier, London UK). A total of 447 articles were then evaluated, in accordance to the PRISMA standards [ 10 ]. In the next step of the screening process, 409 articles were removed due to ire-levance based on titles and abstracts. Finally, 38 full text articles were assessed and checked for eligibility criteria. Following full-text screening, 28 articles (Table 4) were eliminated, as they were not in accordance to inclusion criteria [ 14 - 41 ]. Any inconsistency over final inclusion was discussed and resolved amongst two review authors (IA and SP), whereas, a third reviewer (FK) acted as mediator. Thus, ten full text articles that meteligibility criteria and were included in present systematic review. Detailed summary of data selection was presented in the form of PRISMA 2020 flow diagram (Figure 1).

**Table 4:** The excluded studies are as follows: (EAL: Electronic apex locator, WL: Working length, SD: Standard deviation, CBCT: Cone beam computed tomography, DR: Digital radiography)

Sr. no	Author and Year	Title	Reason for exclusion
1.	Shanmugaraj M, *et al*. (2007) [ 14 ]	Evaluation of working length determination methods: an *in vivo* / *ex vivo* study	They have included permanent teeth with mature apices
2.	Krajczár K, *et al*. (2008) [ 15 ]	Comparison of radiographic and electronical working length determination on palatal and mesio-buccal root canals of extracted upper molars	They have included permanent molar teeth. They haven’t specified gold standard method
3.	Krajczár K, *et al*. (2008) [ 16 ]	Direct comparison of working length determination by ProPex electronic apex locator and radiographic method--an *in vitro* study	They have included permanent molar teeth. They haven’t specified gold standard method
4.	Ravanshad S, *et al*. (2010) [ 17 ]	Effect of working length measurement by electronic apex locator or radiography on the adequacy of final working length: A randomized clinical trial	they have included 20 to 65 years age group patient who presented for endodontic therapy
5.	Cianconi L, *et al*. (2010) [ 18 ]	Accuracy of three electronic apex locators compared with digital radiography: an *ex vivo* study	They selected periodontally involved human teeth extracted from 35- to 60-year-old patients
6.	Real DG, *et al*. (2011) [ 19 ]	Accuracy of working length determination using 3 electronic apex locators and direct digital radiography	Twenty extracted human maxillary premolars were selected
7.	Parekh V, *et al*. (2011) [ 20 ]	Comparative study of periapical radiographic techniques with apex locator for endodontic working length estimation: an *ex vivo* study	They have included premolar teeth
8.	Vieyra JP, *et al*. (2010) [ 21 ]	Comparison of working length determination with radiographs and two electronic apex locators	They have included permanent teeth
9.	Vieyra JP, *et al*. (2011) [ 22 ]	Comparison of working length determination with radiographs and four electronic apex locators	They have included permanent teeth
10.	Saritha S, *et al*. (2012) [ 23 ]	Clinical evaluation of Root ZX II electronic apex locator in primary teeth	They haven’t taken any gold standard method for comparison
11.	Singh SV, *et al*. (2012) [ 24 ]	An *in vivo* comparative evaluation to determine the accuracy of working length between radiographic and electronic apex locators	They included 20 patient aged 25 to 55 years undergoing extraction because of periodontal and orthodontic reasons
12.	Kishor KM (2012) [ 25 ]	Comparison of working length determination using apex locator, conventional radiography and radiovisiography: an *in vitro* study	They have included permanent maxillary central incisors
13.	Mandlik J, *et al*. (2013) [ 26 ]	An *in vivo* evaluation of different methods of working length determination	They have included premolar and supernumerary teeth
14.	Oznurhan F, *et al*. (2014) [ 27 ]	Clinical evaluation of apex locator and radiography in primary teeth	They haven’t taken any gold standard method for comparison
15.	Basso MD, Jeremias F, Cordeiro RC, Santos-Pinto L (2015) [ 28 ]	Digital radiography for determination of primary tooth length: *in vivo* and *ex vivo* studies	They compared accuracy of radiographic tooth length obtained from *in vivo* digital radiograph with that obtained from *ex vivo* digital radiograph
16.	Singh D, *et al*. (2015) [ 29 ]	Comparative evaluation of adequacy of final working length after using Raypex5 or radiography: an *in vivo* study	They included patient aged 20 to 45 years who presented for endodontic therapy
17.	Carneiro JA, *et al*. (2016) [ 30 ]	Comparison of working length determination using apex locator and manual method *ex vivo* study	They have included permanent single rooted teeth. They compared electronic measurement with manual method
18.	Dutta K, *et al*. (2017) [ 31 ]	Comparative evaluation of three methods to measure working length - Manual tactile sensation, digital radiograph, and multi detector computed tomography: An *in vitro* study	They compared working length with three different methods manual tactile sensation, digital radiograph and Multi detector computed tomography.
19.	Khateeb SU, *et al*. (2017) [ 32 ]	Comparative study for determination of toot vanal working length accuracy by different methods–an *in vivo*/*in vitro* study	They used fifty adult human single rooted teeth intended for extraction with mature apices
20.	Adriano LZ, *et al*. (2018) [ 33 ]	*In vitro* comparison between apex locators, direct and radiographic techniques for determining the root canal length in primary teeth	They compared the accuracy of EAL with the conventional radiographic techniques
21.	Rathore K, *et al*. (2020) [ 34 ]	Comparison of accuracy of apex locator with tactile and conventional radiographic method for working length determination in primary and permanent teeth	They compared the apex locator with a conventional radiographic method for working length determination in primary and permanent teeth.
22.	Davalbhakta RN, *et al*. (2021) [ 35 ]	Comparative evaluation of root ZX Mini® apex locator and digital radiography in determining the working length of primary molars: An *in vivo* study	They used tactile method as gold standard method for WL determination
23.	Goel T, *et al*. (2021) [ 36 ]	Comparative evaluation of working length using conventional radiographic method, radiovisiography, and apex locator in single-rooted permanent teeth	They have included permanent single rooted teeth
24.	Mousavi SA, *et al*. (2021) [ 37 ]	Comparative evaluation of root canal working length determination with three methods: conventional radiography, digital radiography and Raypex 6 apex locator: An experimental study	They have included permanent single rooted teeth
25.	Singh AK, *et al*. (2021) [ 38 ]	Evaluation of the efficacy of different systems in determination of root canal working length: A comparative study	They have included premolar teeth
26.	Ramezani M, *et al*. (2022) [ 39 ]	Accuracy of three types of apex locators versus digital periapical radiography for working length determination in maxillary premolars: An *in vitro* study	They have included premolar teeth
27.	Cardoso ML, *et al*. (2022) [ 40 ]	*In vitro* determination of working length in primary teeth	They have not mentioned about mean and SD values and not compared DR with apex locator
28.	Shibin J, *et al*. (2022) [ 41 ]	Evaluation of the working length determination accuracy by cone-beam computed tomography in primary teeth	They have evaluated CBCT, conventional radiography and apex locator. They haven’t used DR

### Study characteristics

The study characteristics of 10 included studies were listed in Table 5. The included studies were published from January 1, 2005 to January 31, 2023. Five of the studies were *in vitro* study [ 42 - 46 ], four were *ex vivo* [ 3 - 4 , 47 - 48 ] and one was *in vivo* study [ 6 ]. No randomized and non-randomized clinical trials were found. The sample size of included studies ranged from 20-90 extracted human primary teeth. Age of the participants in the *in vivo* and *vivo* studies varied from five to fifteen years. EAL used in the intervention group were Formatron D 10, Root ZX, Joypex 5, Root ZX mini, DentaPort ZX, COXO C Smart-1 Pro, iPex, Apex ID and Propex II, whereas DR used were size #0 and size #1. Accuracy was evaluated under stereomicroscopy using direct observation of apical exit of advancing K file, until tip of the file was visible at apical foramen or apical resorption level. Outcomes were assessed by comparing the difference among the actual length, DR and apex locator working length measurement. A statistically insignificant difference between EAL and DR was seen in seven out of 10 articles, whereas, other three studies showed statistically significant difference, indicating that EAL was more closely related to actual working length than DR.

**Table 5:** Data extraction sheet; (ARCL: Actual root canal length, EAL: Electronic apex locator, WL: Working length, SD: Standard deviation, CBCT: Cone beam computed tomography, DR: Digital radiography)

Study ID	Author	Sample	Age group	Intervention	Comparison	Actual WL/ reference method	Outcome	Study design	Result		
									Mean ± SD	*p* value	
1	Subramaniam P *et al*. (2005) [ 14 ]	22, single-rooted primary teeth	-	Formatron D 10 EAL	Digital radiographs with the digital sensor (Cygnus Media, Cygus Technologies, USA)	Actual root canal length (ARCL) of each tooth was measured under stereomicroscopy using direct observation of apical exit of k file	Higher similarity between apex locator measurements and actual canal length, followed by digital radiography and actual canal length, No significant difference was seen.	*In vitro*	EAL=15.94±2.06	*p*> 0.05	
									DR=15.91±1.60		
									ARCL=15.53 ± 2.64		
2	Mello-Moura AC *et al*. (2010) [ 19 ]	20 primary incisors	-	Root ZX apex locator	RVG (Ultimate Image, Trophy, France)	ARCL of each tooth was measured at 15x stereomicroscopy magnification	EAL gave low absolute differences compared with digital radiographic method	*Ex vivo*	Absolute differences among the ARCL, RVG, EAL	*p*< 0.05	
									EAL=0.36 ± 0.30		
									RVG=1.40 ± 2.16		
3	Neena IE *et al*. (2011) [ 6 ]	30 primary teeth,90 canals	5-11 years	Apex locator	DR	Conventional radiographic method	No significant difference in the mean root length measurements in both technique	*In vivo*	Apex locator=11.79±1.70	*p*> 0.05	
									DR=11.98 ±1.70		
									Conventional radiograph=11.76±1.67		
4	Tawil S *et al*. (2012)[ 15 ]	30 Extracted primary incisors	-	Root ZX, J. Morita Corporation, Tokyo, Japan	Digital x-ray sensor size # 1	ARCL was determined by advancing number 15K file until the tip of the file was showed by the naked eye to be with the level of the apical foramen	No significant difference between apex locator and digital X-Ray both showed lowest mean differences from actual length	*In vitro*	Apex locator=12.87±1.84	p> 0.05	
									Digital X-Ray =12.89 ±1.82		
									ARCL =13.07 ±1.78		
5	Wankhade AD *et al*. (2013) [ 4 ]	70 extracted single rooted primary teeth	5-8 years	Joypex 5 (Denjoy Dental Co, Chin)	Size # 0 Schick CDR intraoral X-ray sensor (Schick Technologies, USA)	Stereomicroscopic examination under 8x magnification to determine ARCL	Group 1 (without PRR) EAL revealed statistically insignificant difference when compared with ARCL	*In vitro*	EAL=16.43±0.79	EAL V/S ARCL	DR V/S ARCL
									DR=16.45±0.78	>0.98	<0.97
									ARCL=16.44±0.79		
							Group 2 (with 1/4th PRR) EAL revealed statistically insignificant difference when compared with ARCL		EAL= 13.4±0.79	<0.96	<0.99
									DR= 13.44±0.69		
									ARCL= 13.40±0.72		
							Group 3 (with 1/4th to 3/4th PRR). DR showed a significant difference when compared with ARCL		EAL= 9.47±3.38	>0.99	<0.01
									DR= 14.77±1.33		
									ARCL= 9.47±3.43		
6	Kumar LV *et al*. (2016) [ 3 ]	22 children,41 root canals (seven single-rooted and 15 multi rooted canals)	6-15 years	Root ZX mini apex locator	RVG (Suni Medical Imaging Inc., California, USA)	Actual WL of each canal was measured (the apical exit of the file at the apical foramen or resorption bevel of the root is observed	No significant difference between EAL and DR. Lowest mean difference was observed in the EWL group indicating that the use of EAL consistently brought the file tip closer to the apex	*Ex vivo*	EAL=12.659±1.71	p= 0.609	
									DR=13.037±1.50		
									Actual WL=12.67±1.70		
7	Sahni A *et al*. (2020) [ 16 ]	90 extracted single rooted primary teeth	-	DentaPort ZX (J Morita corp., Kyoto, Japan)	Digital radiograph sensor (Vatech EZ Sensor, Humanray Co. Ltd., Korea)	Actual WL was measured until the tip of the file was just visible at the apex/apical foramen or the apical resorption level.	No significant difference between EAL and DR, Electronic measurement was closer to actual WL as compared to digital radiography	*In vitro*	EAL= 10.10 ± 1.78	p= 0.066	
									DR = 10.08 ± 2.10		
									Actual WL= 10.36 ± 1.80		
8	Kayabasi M *et al*. (2020) [ 17 ]	20 extracted primary molars with resorption and 20 primary molars without resorption-	COXO C Smart-1 Pro, iPex and Apex ID	RVG with a Size#1 sensor (CASTELLINI X-VS CMOS Radiography, Italy)	Actual WL was measured by inserting a size 15 K-file in the canal until the file tip became visible at the apical foramen under 6× magnification using a microscope	No statistically significant difference between the groups with and without root resorption, In the teeth with resorption the nearest measurements to actual WL were Apex ID > COXO C Smart-1 Pro> DR > i Pex respectively. In the teeth without resorption the nearest measurements to actual WL were Apex ID > DR > COXO C Smart-1 Pro = iPex respectively	*In vitro*	Teeth without root resorption	Teeth with root resorption	Teeth without root resorption	Teeth with root resorption
								Actual WL= 11.49±1.63	Actual WL = 9.53±1.44	p= 0.931	p= 0.926
								COXO= 10.92±1.52	COXO= 9.32±1.40		
								iPex= 10.92±1.56	iPex= 9.22±1.38		
								Apex ID= 11.18±1.57	Apex ID= 9.54±1.32		
								DR= 11.03±1.64	DR= 9.24±1.25		
9	Pol DS *et al*. (2021) [ 18 ]	78 canals (30 extracted primary molar)	-	Propex II apex locator	DR (Schick Sirona, Germany)	Actual WL of each tooth was measured at 15X magnification under stereomicroscope	EAL shows highest closer value to actual WL thus provided better performance in working length determination in comparison to DR	*In vitro*	Absolute difference of actual WL	p< 0.05	
									EAL =0.007250± 0.2612		
									DR= 1.9570± 1.553		
10	Khan SA *et al*. (2022) [ 20 ]	84 root canals (58 primary teeth)	4–12 years	Root ZX mini Apex Locator (J Morita Corp, Tokyo, Japa)	DR	Actual WL of each canal was measured using Dental Loupes with 2.5X magnification	Statistically significant difference is seen in all the three groups	*Ex vivo*	Absolute differences among the actual WL, EAL and DR	EAL V/S Actual WL	DR V/S Actual WL
									DR= 0.88± 0.79	0.18	< 0.001
									EAL= ₋0.02 0.12		

### Risk of bias assessment

**Figure 2:**
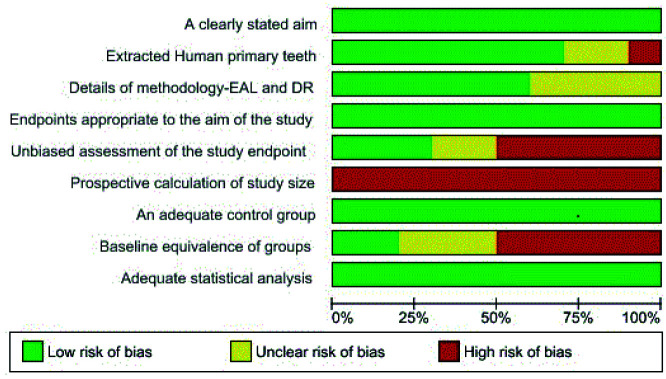
Risk of bias graph: review author’s judgments about each risk of bias item presented as percentages across all included studies

The methodological quality assessment of the selected studies using modified version of MINORS criteria was depicted in Figure 2. It demonstrates the reviewer’s assessment, regarding each risk of bias, which has been presented as percentages across all the included studies. This scale was modified because no clinical trials were found during the search study. Thus, only *in vivo*, *in vitro* and *ex vivo* studies were included. The scores were categorized as low, unclear, and high risk of bias. Low risk of bias indicates plausible bias that was not likely to change the results seriously, unclear risk of bias indicates bias that raised doubts about the results, and high risk of bias indicates bias that does not inspire confidence in the results.

The summary of risk of bias presented in Table 6 show, seven studies by Mello Moura *et al*. [ 47 ], Wankhade *et al*. [ 4 ], Kumar *et al*. [ 3 ], Sahni *et al*. [ 44 ], Kayabasi *et al*. [ 45 ], Pol *et al*. [ 46 ] and Khan *et al*. [ 48 ], that presented low risk of bias which indicates high quality of evidence.

**Table 6:** Summary of risk of bias (MINORS: Methodological Index for Non-Randomized Studies, EAL: Electronic apex locator, DR: Digital Radiography)

S. no.	MINORS criteria	Scoring*									
		Study ID 1	Study ID 2	Study ID 3	Study ID 4	Study ID 5	Study ID 6	Study ID 7	Study ID 8	Study ID 9	Study ID 10
1	A clearly stated aim	2	2	2	2	2	2	2	2	2	2
2	Extracted Human primary teeth	0	2	1	1	2	2	2	2	2	2
3	Details of methodology-EAL and DR	1	2	1	2	1	2	2	2	2	1
4	Endpoints appropriate to the aim of the study	2	2	2	2	2	2	2	2	2	2
5	Unbiased assessment of the study endpoint	1	2	0	0	1	2	0	0	0	2
6	Prospective calculation of study size	0	0	0	0	0	0	0	0	0	0
7	An adequate control group	2	2	2	2	2	2	2	2	2	2
8	Baseline equivalence of groups	0	0	0	0	0	2	1	1	2	1
9	Adequate statistical analysis	2	2	2	2	2	2	2	2	2	2
Total score (out of 18)		10	14	10	11	12	16	13	13	14	14
Grading#		High risk	Low risk	High risk	High risk	Low risk	Low risk	Low risk	Low risk	Low risk	Low risk

* The items are scored 0 (not reported), 1 (reported but inadequate) or 2 (reported and adequate).

# GRADING: Low risk: score equal to 12 or greater than 12; High risk: score less than 12

The remaining three studies conducted by Subramaniam *et al*. [ 42 ], Neena IE *et al*. [ 6 ], and Sherif *et al*. [ 43 ], showed high risk of bias, which indicates low quality of evidence. The summarized findings show that the included studies are of moderate quality overall, with a high risk of bias evident only at definite points.

## Discussion

Early loss of primary teeth can cause space closure resulting in malocclusion of permanent dentition. Pulpectomy procedure involves complete removal of both coronal and radicular pulp. WL determination is an essential step in pulpectomy procedure, as it decides the position of apical foramen and thus the extent of obturation.

It is hard to judge the root canal anatomy of primary teeth because of continuous ongoing resorption and root canal shape. [ 49 ] The position of canal terminus and measurement of WL can be done by various techniques.

One of the widely used methods is the radiographic method for WL determination. However, measurement by this method is generally one or a half-millimeter (mm) short of the radiographic apex, where the apical constriction is generally thought to be located [ 50 ]. To eliminate the many problems associated with the radiographic methods, apex locator is now being used clinically and has become an essential part of the armamentarium of root canal procedure. Several studies have compared WL determination done by EAL and DR in primary teeth; only nine studies have been considered for inclusion in this systematic review. 

The sample size is important to make any inferences about a population from a sample. Sample size was 20 to 90 primary teeth for the included studies. None of the studies revealed any details about the sample size calculation. This could lead to an increased chance of risk of bias of individual studies. The amount of physiologic root resorption can affect the determination of WL in an endodontic procedure. Amount of root resorption was reported in majority of the studies, however, three studies, Subramaniam P *et al*. [ 42 ], Neena IE *et al*. [ 6 ] and Sherif B *et al*. [ 43 ], inadequately reported the amount of root resorption. The threshold file size for working length measurements should be size # 15 K, as the tip diameter of the No # 10K files is less than 120 micrometers, which is not identifiable [ 6 ]. This finding was in accordance with studies by Kumar L V *et al*. [ 3 ], Sherif B *et al*. [ 43 ] and Sahni A *et al*. [ 44 ], Kayabasi M *et al*. [ 45 ]. Density profile plot analysis for digital images found that size #20 files were employed for radiographic length computation, because size #15 and size #10 files have decreasing perceptibility of file length [ 51 ].

Because of anatomical variances, anatomical structure interference, and projection problems, determining the precise radiographic root canal length is difficult [ 52 ]. Kumar L V *et al*. [ 3 ], Mello Moura *et al*. [ 47 ], Neena IE *et al*. [ 6 ], Sherif B *et al*. [ 43 ] and Kayabasi M *et al*. [ 45 ] employed the paralleling technique which is difficult to perform on paediatric patients. However, in the remaining four studies, they have not reported about the projection technique. These variables may result in over-instrumentation and over-filling of canals, causing permanent tooth bud injury [ 1 , 53 ].

As calibrated digital measurements are more accurate than un-calibrated ones [ 54 ], digital image calibration was performed, prior to WL determination, using an on-screen calibration tool. Kim *et al*. [ 55 ] demonstrated in their study that radiographic measurement of WL using an onscreen straight-line measurement was effective.

Apex locators are classified based on their generations. The first and second generations of EALs are obsolete, and are no longer manufactured or utilized in the modern era of dentistry. Nasiri *et al*. [ 56 ] performed a systematic review and meta-analysis and found that all generations are equally beneficial and accurate in WL evaluation.

The real WL was calculated by subtracting 0.5mm from the distance between the apical foramen and the apical constriction, which is roughly 0.5-1.0 mm [ 57 ]. A 0.5 mm margin was employed in numerous researches evaluating the accuracy of WL. Amongst the included studies, five studies, by Kumar L V *et al*. [ 3 ], Sahni *et al*. [ 44 ], Kayabasi M *et al*. [ 45 ], Pol D S *et al*. [ 46 ] and Khan SA *et al*. [ 48 ], determined WL by subtracting 0.5mm from apical foramen, while five studies by Wankhade AD *et al*. [ 4 ], Neena IE *et al*. [ 6 ], Subramaniam P *et al*. [ 42 ], Sherif B *et al*. [ 43 ] and Mello Moura *et al*. [ 47 ] did not report about the same. When compared to DR, it was found that the WL measurement provided by the EAL was near to the real WL determination in all of the included studies. This was in line with a number of research studies [ 42 , 58 ] that measured the precision of the apex locator in primary teeth. The radiographic length determination is difficult in primary teeth because of constant change in apex position due to continuous ongoing root resorption. However, only a few studies have found that in roots with wider apical foramen, EAL measurements are much shorter than the true WL [ 59 ]. In primary dentition, however, most studies found EAL accuracy rates of 64 to 96 percent [ 58 , 60 ]. In a study by Sahni *et al*. [ 44 ], a substantial correlation was discovered between the WL of the reference technique and EAL, and this was in agreement with Shabahang *et al*. [ 61 ], who concluded that EAL reliably determines the root end even in cases when root resorption is present. Furthermore, this is consistent with the findings of other researchers who reported the great accuracy of EALs in primary teeth. 

The disparity in accuracy of WL may be due to variation in study designs, sample sizes, rate of resorption, file size, reference point, radiographic technique, and different generations of EAL used in the included studies.

This review has some limitations, one of which was usage of limited databases for search strategy. Another limitation was use of English literature only as, articles published in other languages were excluded. Finally, caution should be exercised before applying the results of this review in a clinical scenario, as all the included studies were of *in vitro* design. 

## Conclusion

Within the limitation of present systematic review, we found moderate quality of evidence to suggest accuracy of EAL and DR for WL determination. Majority of studies showed statistically insignificant difference between EAL and DR. However, in few studies, electronic measurement was close to actual WL as compared to DR. Based on the evidence that is currently available, EAL can be considered as an alternative for working length measurement in primary teeth. However, future research should include information about the study populations, blinding and sufficient documentation.
